# Supplementary material for: Causal interplay between lactose intolerance and gut microbiota: a combined bidirectional Mendelian randomization and in vivo validation study
Source: Front Nutr. 2026 Jun 1;13:1803337. doi: 10.3389/fnut.2026.1803337 (PMC13265576; doi:10.3389/fnut.2026.1803337)
Supplement: Supplementary file 1 [file Data_Sheet_1.zip › supplementary materials/Forward/forest plot/ebi-a-GCST90027640.finngen_R12_E4_LACTONAS.pdf]

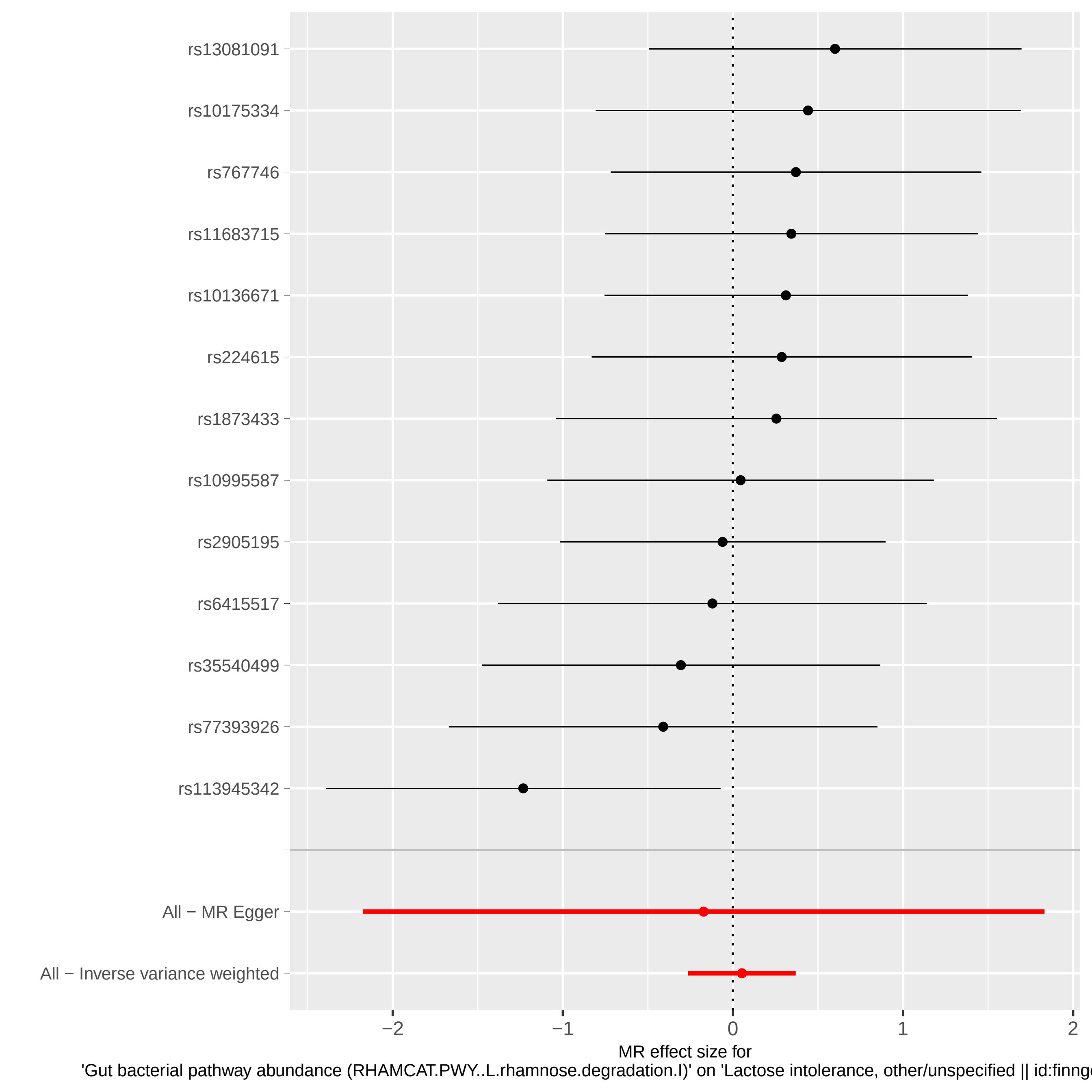

rs13081091
rs10175334
rs767746
rs11683715
rs10136671
rs224615
rs1873433
rs10995587
rs2905195
rs6415517
rs35540499
rs77393926
rs113945342
All − MR Egger
All − Inverse variance weighted
−2
−1
0
1
2
MR effect size for
'Gut bacterial pathway abundance (RHAMCAT.PWY..L.rhamnose.degradation.I)' on 'Lactose intolerance, other/unspecified || id:finng
